# Supplementary material for: Mechanisms governing the pioneering and redistribution capabilities of the non-classical pioneer PU.1
Source: Nat Commun. 2020 Jan 21;11:402. doi: 10.1038/s41467-019-13960-2 (PMC6972792; doi:10.1038/s41467-019-13960-2)
Supplement: Supplementary file 7 — Source data [file 41467_2019_13960_MOESM7_ESM.zip › Source_Data/Figure6/Figure6B_MotifScanOutput/ETS-specific/homerResults/motif10.similar.html]

motif10

## Information for motif10

A
G
T
C
G
A
C
T
A
C
G
T
G
A
C
T
A
T
C
G
C
G
T
A
C
G
A
T
T
A
C
G
G
C
A
T
A
C
T
G
  
Reverse Opposite:  

T
G
A
C
C
G
T
A
A
T
G
C
G
C
T
A
C
G
A
T
T
A
G
C
C
T
G
A
T
G
C
A
C
T
G
A
T
C
A
G
  

|  |  |
| --- | --- |
| p-value: | 1e-110 |
| log p-value: | -2.551e+02 |
| Information Content per bp: | 1.653 |
| Number of Target Sequences with motif | 1234.0 |
| Percentage of Target Sequences with motif | 16.12% |
| Number of Background Sequences with motif | 3042.4 |
| Percentage of Background Sequences with motif | 7.54% |
| Average Position of motif in Targets | 102.9 +/- 53.4bp |
| Average Position of motif in Background | 99.8 +/- 61.7bp |
| Strand Bias (log2 ratio + to - strand density) | 0.0 |
| Multiplicity (# of sites on avg that occur together) | 1.11 |
| Motif File: | file (matrix) reverse opposite |

### Similar de novo motifs found

|  |  |  |  |  |  |  |  |
| --- | --- | --- | --- | --- | --- | --- | --- |
| Rank | Match Score | Redundant Motif | P-value | log P-value | % of Targets | % of Background | Motif file |
| 1 | 0.971 | G C T A A T G C G C T A C G A T T A G C C G T A T G C A C G T A A T C G T A C G C T A G | 1e-99 | -228.292958 | 12.37% | 5.31% | motif file (matrix) |
| 2 | 0.963 | C G T A A T G C C G A T C G A T T A G C C G T A T G C A C G T A A T C G | 1e-96 | -222.830230 | 14.11% | 6.57% | motif file (matrix) |
| 3 | 0.929 | C A G T A C G T A G T C A G T C A C G T A C G T A C G T C A T G C G T A C G A T T A C G C G A T C G A T | 1e-93 | -216.208808 | 16.51% | 8.39% | motif file (matrix) |
| 4 | 0.863 | A G T C A C G T A C G T A C G T A T C G C G T A C G A T T A G C | 1e-64 | -149.023618 | 8.06% | 3.42% | motif file (matrix) |
| 5 | 0.904 | A G T C A C G T A C G T A C G T A T C G C G T A C G A T | 1e-60 | -138.769154 | 13.01% | 7.08% | motif file (matrix) |
| 6 | 0.854 | C G T A T A C G G T C A C G A T T A G C G C T A T C G A C T G A C T A G T A C G T C G A G T A C | 1e-47 | -109.496380 | 4.00% | 1.32% | motif file (matrix) |
| 7 | 0.799 | A C T G A G T C A C G T A C G T A C G T A C T G G T C A C G T A T C A G G C T A A G C T C G T A | 1e-40 | -92.546983 | 10.44% | 6.02% | motif file (matrix) |
| 8 | 0.768 | A G T C A C G T C G A T A C G T T A C G G T C A G A C T A G T C C G A T A G C T G T C A C G T A G A C T G A T C | 1e-15 | -34.834325 | 0.31% | 0.01% | motif file (matrix) |
